# Selenium, Selenoproteins, and Immunity

**DOI:** 10.3390/nu10091203

**Published:** 2018-09-01

**Authors:** Joseph C. Avery, Peter R. Hoffmann

**Affiliations:** Department of Cell and Molecular Biology, John A. Burns School of Medicine, University of Hawaii, 651 Ilalo Street, Honolulu, HI 96813, USA; jcavery@hawaii.edu

**Keywords:** selenocysteine, macrophage, T cell, antibody, inflammation, cancer

## Abstract

Selenium is an essential micronutrient that plays a crucial role in development and a wide variety of physiological processes including effect immune responses. The immune system relies on adequate dietary selenium intake and this nutrient exerts its biological effects mostly through its incorporation into selenoproteins. The selenoproteome contains 25 members in humans that exhibit a wide variety of functions. The development of high-throughput omic approaches and novel bioinformatics tools has led to new insights regarding the effects of selenium and selenoproteins in human immuno-biology. Equally important are the innovative experimental systems that have emerged to interrogate molecular mechanisms underlying those effects. This review presents a summary of the current understanding of the role of selenium and selenoproteins in regulating immune cell functions and how dysregulation of these processes may lead to inflammation or immune-related diseases.

## 1. Introduction

Selenium was discovered by the Swedish chemist Jöns Jakob Berzelius in 1817 and was considered a toxic element for humans and livestock for nearly 150 years [1]. However, in 1957, the benefits of selenium for humans and other mammals were revealed in landmark studies by Klaus Schwartz and Calvin Foltz who demonstrated that dietary selenium protected rats against liver necrosis [2]. Since then, the role of selenium as a trace mineral nutrient in human health and the mechanisms by which it exerts its biological effects have become better understood. Adequate levels of bioavailable selenium are functionally important for several aspects of human biology including the central nervous system, the male reproductive biology, the endocrine system, muscle function, the cardiovascular system, and immunity [3,4]. Many pathological conditions involving the immune system can be affected by the selenium status in an individual, which can be influenced by several factors such as the levels and forms of selenium ingested, the conversion of selenium compounds into metabolites, and genetic characteristics that can impact the use of these metabolites. Selenium deficiency is rare in the United States and Canada [5], but regions of China, New Zealand, and parts of Europe and Russia have low levels of selenium in soil and food [6]. The extent to which immune-related diseases are impacted by differences in selenium intake and how supplementation approaches may be utilized to mitigate these health issues is not entirely clear. However, the development of new high-throughput omic approaches and bioinformatics tools have improved our understanding of the effects of selenium immuno-biology in humans. Additionally, novel experimental systems have provided valuable insight into mechanisms underlying those effects.

The U.S. recommended dietary allowance for selenium for adults is 55 μg/day and most individuals achieve this level while several other countries have higher recommended allowances due to a lower average selenium status in their populations [7]. For example, adults in the U.K. are recommended to ingest 60 μg/day for adult women and 75 μg/day for lactating women and adult men [8]. Commonly used measures of a selenium status include plasma and serum selenium concentrations as well as selenoprotein P levels and glutathione peroxidase activity [9,10]. The average plasma selenium concentration in the U.S. is 70 ng/mL, which is relatively high with selenium intake found to be lower in areas within China and Europe, in New Zealand, and in other parts of the world [11,12]. Dietary selenium is obtained through a wide variety of foods including grains, vegetables, seafood, meat, dairy products, and nuts [13]. The predominant form of selenium ingested by humans is selenomethionine. However, other forms of selenium are also present in foods. Selenium gets metabolized into various small molecular weight seleno-compounds including some that may exert biological effects through redox reactions that can affect cellular processes like DNA repair and epigenetics [14,15]. These bioactive metabolites include hydrogen selenide and methylated selenium compounds like methylseleninic acid, which exerts chemo-preventive effects [16]. Most of the effects of dietary selenium on immune functions are attributable to the insertion of this element into a family of proteins called seleno-proteins. What separates selenium from other nutritional elements is the fact that it is incorporated directly into proteins as the 21st amino acid, selenocysteine (Sec). The synthesis of selenoproteins within cells requires a dedicated set of protein and tRNA factors assembled on ribosomes along with the selenoprotein mRNA, which contains unique structural elements. The coordinated interaction of these elements leads to co-translational insertion of Sec into the nascent polypeptide when the ribosome encounters a uridine-guanosine-adenosine (UGA) codon, which is typically used as a stop codon in other mRNAs [17]. Under conditions of low selenium status, this translational process stalls at the UGA codon and both the mRNA and truncated protein may get degraded through two separate processes called nonsense-mediated decay (NMD) and destruction via C-end degrons (DesCEND), respectively [18,19]. Certain mRNA characteristics potentially play a role in NMD sensitivity such as the location of the Sec codon (UGA) relative to exon–exon junctions [18]. Therefore, the selenium status is directly related to levels of different selenoproteins in different tissues. Given the combined effects of NMD and DesCEND, there appears to be a hierarchy of selenoprotein synthesis that results in some family members having a higher priority of expression under selenium-limiting conditions [20]. In addition, certain tissues like the brain, endocrine tissues, and testes retain selenium under deficient conditions shed light on the priorities given to different physiological systems when selenium levels are low.

In humans, 25 selenoproteins have been identified and 24 of those exist as Sec-containing proteins in rodents [21], which highlights the value of rodent models for determining roles for members of this protein family in immune responses. Selenoproteins exhibit a wide variety of tissue distribution and functions [17]. While many members of the selenoprotein family function as enzymes involved in redox reactions, some are likely not enzymes themselves and functions are gradually becoming better understood for these non-enzymatic members. The most completely characterized selenoprotein enzymes related to immune functions include glutathione peroxidases (GPXs), thioredoxin reductases (TXNRDs), iodothyronine deiodinases (DIOs), methionine-*R*-sulfoxide reductase B1 (MSRB1), and selenophosphate synthetase 2 (SPS2). For non-enzymatic selenoproteins, the best characterized in terms of immune cell function is selenoprotein K (SELENOK). Table 1 lists selenoproteins and their functions and a more detailed discussion of roles for individual selenoproteins in different immune cells and tissues is provided below.

As mentioned above, nearly all tissues are affected by changes in the selenium status or selenoprotein expression. While the focus of this review is on the immune system, it is important to first touch on other physiological systems impacted by the levels of selenium and selenoproteins. Embryonic lethality arising from deletion of the *trsp* gene encoding the Sec-tRNA required for translation [54] demonstrates the essential nature of selenoproteins. In fact, there have been four individual selenoprotein knockout mice in which gene ablation was shown to result in embryonic lethality: GPX4, TXNRD1 and 2, and Selenoprotein T (SELENOT) [32,51,55,56]. An essential role for one of these selenoproteins in the area of development was demonstrated by the recent study, which showed that GPX4 protects a critical population of interneurons from ferroptotic cell death [29]. In the muscular system, genetic maladies involving selenoproteins include multi-minicore diseases (MmD) such as rigid spine syndrome (RSS) resulting from mutations in the human gene encoding Selenoprotein N (SELENON) [57,58] and an associative dysfunction of the ryanodine receptor 1 (RyR1) receptor [59]. Transgenic overexpression of some selenoproteins potentially regenerates wasted muscle in mice [60]. Thyroid hormone metabolism is dependent upon the combined actions of the three selenoproteins known as iodothyronine deiodinases 1-3 (DIO1-3) [61]. Thus, selenium deficiencies can affect thyroid gland function and the many physiological systems impacted by thyroid hormone activity. In the hepatic system, selenium is absorbed from the gastrointestinal tract and utilized for biosynthesis of selenoproteins including Selenoprotein P (SELENOP), which is the primary plasma selenium transport protein [62]. Several groups have observed that SELENOP inactivation results in normal hepatic selenium levels while selenium content in other tissues decreases significantly. This reduces the total GPX and TXNRD pools [63,64]. Consequently, those organs that rely on SELENOP-mediated selenium delivery become deficient when some tissues are given ‘priority’ over others for retention of this element since delivery through SELENOP decreases.

The central nervous system is appreciably dependent on an adequate selenium supply and, as mentioned above, diets that are slightly deficient in selenium do not elicit neurological deficits due to the preservation of selenium content in the central nervous tissue during dietary selenium restriction [65]. On the other hand, a targeted reduction in brain selenium reduces SELENOP bioavailability and causes spontaneous neurological deficits [66], which are reversed by selenium supplementation [67,68]. Additionally, overexpression of TRX1 has been found to mitigate oxidative challenges in the brain [69]. GPX1 was the first mammalian selenoprotein to be discovered [70,71] and has been shown to protect the brain from oxidative insults. Like GPX1, GPX4 protects cortical neurons from exogenous oxidative stress-inducing agents [72,73]. Importantly, the protein oxidation product methionine-*R*-sulfoxide contributes to neurodegenerative diseases and can be repaired by thioredoxin-dependent selenoenzyme MSRB1, which reduces methionine-*R*-sulfoxide back to methionine [36]. Inactivation of MSRB1, however, does not produce neurological deficits [36]. In the kidney, several studies have identified the expression of DIOs, thioredoxin reductases (TRs), and GPXs, but their respective roles have not been fully elucidated. Burk et al. demonstrated that glutathione (GSH) deprivation causes severe pathogenic nephropathy [74] while podocyte-specific ablation of the *trsp* gene in diabetic mice did not enhance markers of nephropathic disease. Moreover, murine renal expression of GPX1 has been reported not to be protective against diabetic nephropathy.

For clinically diagnosed disorders, Keshan disease (KD) is perhaps the most firmly established selenium deficiency-based pathology. This cardiomyopathy was first described in rural areas of China due to low selenium content in foods [75]. There is evidence in mouse models that selenium deficiency promotes the conversion of nonvirulent coxsackievirus B3 strains into a more virulent strain due to an increased oxidative stress [76], which suggests that this infectious agent may be a cofactor. Selenoprotein deficiency may also promote osteochondral diseases including Kashin-Beck disease (KBD). This disease is a poly-pathogenic, degenerative osteochondropathy leading to chondrocyte necrosis [77] and apoptosis [78,79,80], which results in growth retardation and secondary osteoarthrosis [81]. KBD is mainly endemic to Tibet, China, Siberia, and North Korea and is caused in part by poor selenium levels in soil that usually affects children between the ages of 5 to 15 [81,82]. In 1998, Moreno-Reyes et al. established the relationship between this osteoarthropathy and selenium deficiency in rural Tibet [82].

## 2. Selenium and Immunobiology

The importance of adequate levels of dietary selenium and its efficient incorporation into selenoproteins in immunity has been demonstrated in cell culture models, in rodent models, in livestock and poultry studies, and in humans. Selenium deficiency can give rise to immune-incompetence that leads to increased susceptibility to infections and possibly to cancers. There is some evidence that selenium can modulate the pathology that accompanies chronic inflammatory diseases in the gut and liver as well as in inflammation-associated cancers [83,84]. Selenium deficiency and suppressed selenoprotein expression have been implicated in higher levels of inflammatory cytokines in a variety of tissues including the gastrointestinal tract [85,86], the uterus [87], mammary gland tissues [88], and others. However, some inflammatory processes actually increase when selenium intake changes from deficient to sufficient levels. For example, a mouse model of allergic asthma showed that selenium deficiency reduced airway inflammation while adequate selenium intake produced higher levels of inflammation that were then decreased when supra-nutritional levels of selenium were used [89]. In addition, increasing the selenium status through dietary delivery of sodium selenite raised expression levels and translation of mRNAs encoding stress-related selenoproteins as well as genes involved in inflammation and interferon γ IFNγ responses [90].

Selenium supplementation, for the most part, is immuno-stimulatory, which is measured by a wide range of parameters including T cell proliferation, NK cell activity, innate immune cell functions, and many others [91]. This depends on the baseline selenium status and the strongest effects can be seen when supplementation boosts selenium levels from inadequate to adequate while the benefits of increasing an adequate selenium level to supra-nutritional levels is less clear. The activation of human blood leukocytes has been shown to increase in response to selenium-enriched foods [92]. Vaccine responses against pathogens such as poliovirus have been shown to improve with selenium supplementation [93] even though results were mixed when analyzing the influenza vaccine in older adults [94]. Similarly, integrated-omics analyses of pathways affected by the selenium status in rectal biopsies from 22 healthy adults showed reduced inflammatory and immune responses and cytoskeleton remodeling in the suboptimal selenium status group [95]. Similarly, selenium supplementation was shown to modulate the inflammatory response in respiratory distress syndrome patients by restoring the antioxidant capacity of the lungs, which moderated the inflammatory responses through interleukin (IL)-1β and IL-6 levels and meaningfully improved the respiratory mechanics [96].

**Figure 1:**
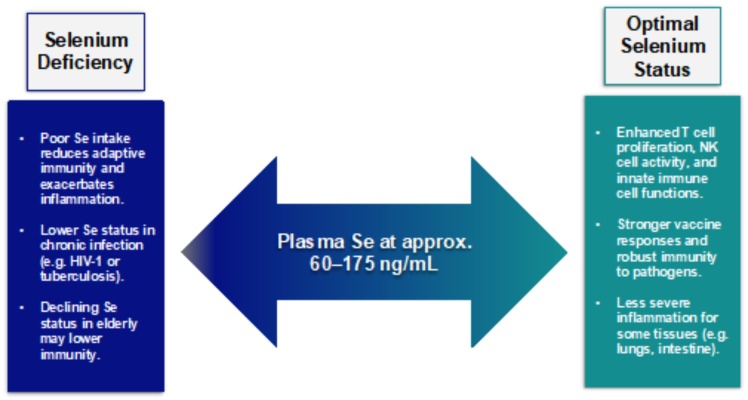
A summary of selenium and immune responses.

There have been few definitive reports of selenium and selenoprotein levels affecting hematopoiesis and the development of the immune system. The deletion of the essential selenoprotein, TXNRD2, does not impair lymphocyte development and maintenance [97]. However, a T cell-specific knockout of all selenoproteins was found to reduce the number of mature T cells emerging from lymphoid tissues [98]. Autoimmunity is an important issue related to immune system development and there have been reports in humans of an increase in the prevalence of autoimmune thyroiditis in low-selenium regions that are consistent with studies in mice [99,100]. While selenium is vital for many immune cell functions (Figure 1), the benefits of applying wide scale selenium supplementation as an approach to boost immunity in the general population have lacked definitive support over the years. This suggests a need for a more refined evaluation of how selenium affects different types of immune responses along with a deeper mechanistic understanding. In the following sections, the effects of selenium on various aspects of immunity and its mechanisms of action will be discussed in further detail.

## 3. Leukocyte Functions

Adaptive immunity is affected by selenium intake including the activation and functions of T and B cells. One immunological feature of selenium levels in vivo is the positive effect that higher selenium has on the proliferation and differentiation of cluster of differentiation(CD)4^+^ T helper (Th) cells. There are several reports of the skewing of T cell immunity toward Th1 phenotypes. For example, our laboratory used a mouse model of viral antigen vaccination to test effects of low (0.087 ppm), medium (0.25 ppm), and high (1.0 ppm) selenium diets and found that Th1 immunity was enhanced along with the T cell receptor signal strength [101]. In a separate study, oral administration of synthetic selenium nanoparticles induced a robust Th1 cytokine pattern after a hepatitis B surface antigen vaccination in a mouse model [102]. Less information is available regarding the effects of selenium on cytotoxic CD8^+^ T cells even though cytotoxic T cells from aged mice (24 months old) showed enhanced mitogen-induced proliferation when treated with selenium supplementation [103]. Mouse knockout models have shown roles for selenoproteins in antibody production. In particular, T cell deletion of the *trsp* gene responsible for the synthesis of all selenoproteins not only affected T cell maturation and activation but reduced the T cell ‘help’ provided to B cells for secreting antibodies, which is determined by low levels of serum immunoglobulin [98]. A small study in humans showed a positive effect on antibody titers against the diphtheria vaccine with selenium supplementation that correspond to increased lymphocyte counts [104]. In a more recent study involving Selenoprotein F (SELENOF) knockout mice, elevated levels of immunoglobulins were detected in the sera that were nonfunctional [38]. The authors of this study concluded that SELENOF functions as a gatekeeper of immunoglobulins in the endoplasmic reticulum (ER), which supports the redox quality control of these proteins and likely other proteins.

Innate immune cell functions have also been shown to be impacted by selenium levels. Macrophages are affected by selenium levels in terms of their inflammatory signaling capacity and anti-pathogen activities. Activation of macrophages through pathogen-associated molecular patterns like lipopolysaccharide (LPS) generates an oxidative burst. Additionally, macrophage activation involves the release of cytokine mediators and arachidonic acid-derived prostaglandins like prostaglandin E2 (PGE2), thromboxane A2 (TXA2), and prostaglandin D2 (PGD2) as well as its metabolite 15-Deoxy-Delta-12,14-prostaglandin J2 (15d-PGJ2). It was shown that selenium induces a phenotypic switch in macrophage activation from a classically activated, pro-inflammatory phenotype (M1) toward an alternatively activated, anti-inflammatory phenotype (M2) [105]. Regarding the latter phenotype, selenium was shown to be pivotal for cyclooxygenase-dependent 15d-PGJ2 generation and M2-mediated clearance of helminthic parasite infections [106]. Evidence from several studies demonstrated that selenium levels and selenoproteins regulate migration and phagocytosis functions in macrophages [107,108]. Experiments involving golden Syrian hamster macrophages and *Staphylococcus aureus* showed that higher levels of selenium in culture media led to significant increases in macrophage phagocytic activity, nitric oxide production, and *S. aureus* killing [109]. Furthermore, pre-treatment of RAW264.7 mouse macrophages with selenium supplementation prior to exposure to *S. aureus* led to lower levels of nuclear factor kappa-light-chain-enhancer of activated B cells (NF-κB) activation and downstream inflammatory cytokine release [110]. Less information is available regarding selenium levels and neutrophil function. However, one study demonstrated that increased selenium intake may protect neutrophils from endogenous oxidative stress [111]. Natural Killer (NK) cells are impacted by dietary selenium intake both directly and indirectly. Serum selenium concentration was positively associated with peripheral CD16^+^ NK cells in older humans [112]. However, functional capacity of these NK cells, e.g., cytotoxicity, was not determined. A separate study in mice showed that selenium supplementation increased the cytotoxic functions of NK cells [113]. The inhibitory receptor, CD94/Natural Killer G2A (NKG2A), was found to be sensitive to selenite treatment, which indicated that NK cell activity may be indirectly increased using this form of selenium [114]. The use of selenium to increase innate immunity may be enhanced when provided along with other nutritional antioxidants. This was demonstrated in prematurely aging mice that exhibited improved macrophage and NK cell functions with a cocktail of antioxidants that included selenium [115].

## 4. Immune Responses to Pathogens

Innate and adaptive immune responses against bacterial and parasitic infections rely on sufficient selenium for eliminating these pathogens. For example, selenium deficiency in mice was shown to impair innate immunity and induce susceptibility to *Listeria monocytogenes* infection [116]. In this study, C57BL/6 mice fed adequate or deficient selenium diets were infected with *L. monocytogenes* and it was found that mice maintained on a selenium deficient diet produced less IFNγ when compared to mice that were fed the control diet. In addition, selenium supplementation decreased the parasitemia of pregnant Wistar rats infected with *Trypanosoma cruzi* [117]. Selenium intake also affects the plasticity of macrophages during immune responses to helminthic parasite infections. For example, a mouse model of infection with *Nippostrongylus brasiliensis*, which is a gastrointestinal nematode parasite, showed that higher levels of dietary selenium led to optimal expression of selenoproteins and selenium-dependent production of cyclooxygenase (COX)-derived endogenous prostanoids crucial for eliminating *N. brasiliensis* infection [118]. Similarly, mice infected with *Heligmosomoides bakeri* required sufficient selenium intake to eliminate these helminthic pathogens and this correlated with increased local expression of Th2-associated genes in infected small intestinal tissues [119].

Bacterial infections in neonates may be a particularly important outcome related to maternal selenium status, which is suggested by studies in humans and rodent models [120,121,122]. These studies do not necessarily distinguish between the effects of the selenium on the immune system and other effects that selenium can have on infant health. In addition to the role of the selenium status on the immune system, one must keep in mind the direct effects that selenium has on bacterial pathogens as well the fact that many bacterial species express selenoproteins [123].

Selenium is one of many nutrients implicated in the severity and progression of tuberculosis (TB) caused by the bacterium *Mycobacterium tuberculosis* [124]. Pulmonary TB patients have lower selenium statuses when compared to healthy controls [125]. Interestingly, investigators also observed that TB patients with concomitant HIV infection exhibited a significantly lower concentration of serum selenium along with augmented wasting versus those without HIV infection. Intensified wasting in TB patients was positively correlated with the severity of lung disease and was associated with low serum selenium levels [126]. Following a two-month intervention study, selenium plus vitamin E supplementation enhanced total antioxidant capacity in patients with pulmonary TB even though the effects on the immune system were not determined [127]. However, several researchers have pointed out that some trace nutrients that may be used as supplements to restore immunity and lung function may also be exploited by *M*. *tuberculosis* to promote growth of the pathogen [128]. This has been supported by data involving the growth of this bacteria under different selenium concentrations [129].

The beneficial effects of a higher selenium status have been supported for some viral infections even though there are some studies that do not conclusively demonstrate effective improvements in anti-viral immunity [130]. Moreover, the antioxidant properties of some selenoproteins have been suggested to contribute to boosting anti-viral immunity [131]. However, some selenoproteins that have not been established as antioxidant enzymes like SELENOK can also play key roles in protecting against viruses [42]. The chronic hepatitis C virus (HCV) has been shown to influence oxidative stress levels in humans and an association between HCV load and the selenium status has been associated with a documented selenium status [132]. Oxidative stress can have genomic altering effects on RNA viruses that can lead to higher virulence of certain viruses themselves and this has been shown to involve the selenium status in the case of coxsackievirus B3 [133]. Thus, the effects of selenium on the virus in some cases may compound the influence of this micronutrient on the immune system. Targeting individuals with low selenium intake or the elderly with a declining selenium status with selenium supplementation may be an effective public health initiative for increasing vaccine responses to viruses [134]. There is evidence to support a positive effect on adaptive immune responses to vaccination against viral pathogens. This causes polio and influenza in populations with a low baseline selenium status [93,135].

The most compelling data available regarding the role of selenium in anti-viral immunity are those related to HIV infection, which is a global pandemic that particularly afflicts persons with inadequate nutrition and directly impairs immunity [136]. Selenium is one micronutrient implicated in disease progression. Low selenium intake has been associated with HIV prevalence [137,138] and the status of CD4^+^ T cell numbers has been correlated with selenium levels in HIV^+^ patients [139]. There is some evidence that selenium malabsorption or overutilization in Acquired immune deficiency syndrome (AIDS) patients may affect or be affected by disease progression [140,141]. In particular, selenium-deficient HIV^+^ patients tend to present with disrupted hemodynamics such as depressed selenium plasma and erythrocyte levels, diminished glutathione peroxidase activity, and stunted cardiac selenium bioavailability. A plasma selenium status is conventionally assessed by SELENOP levels and GPX activity as well as selenium levels, which respond differently to changes in selenium consumption [142]. Thus, it is difficult to directly compare studies using different selenium status readouts [143]. Anti-retroviral therapies may also confound the selenium status [144,145]. However, some studies have not supported this notion [146]. Additionally, selenium is often combined with other nutrients for intervention studies, which makes assessment of its impact difficult to distinguish from other nutritional components. Several cohort studies have illustrated an association between selenium deficiency and progression to AIDS-related mortality [147]. Remarkably, randomized controlled trials demonstrated that selenium supplementation minimized hospitalizations and diarrheal morbidity and improved CD4^+^ T cell counts [141,148]. Similarly, an inhibitory effect of selenium on HIV in vitro due to the radical scavenger effects of glutathione peroxidase has been reported [141]. Glutathione peroxidase and other antioxidant selenoenzymes along with catalase have been implicated in decreasing a viral activation impact on redox control [141,149]. Thus, the potential benefits of selenium supplementation for HIV infection likely resides in the redox regulating selenoenzymes and resides less with the pro-oxidant seleno-metabolites that are found to affect cancer.

## 5. Selenium and Its Effects on a Shift toward Anti-Cancer Immunity

The effects of the selenium status on carcinogenesis or tumor progression have been intensely studied and results have led to a wide variety of conclusions. In humans, there have been several epidemiological studies as well as intervention studies involving different types of cancer, which suggests beneficial effects of higher selenium status [150,151,152]. On the other hand, the selenium status was not found to be a factor in cancer progression in a number of other studies [153,154,155,156]. From the perspective of research in humans, it has proven difficult to separate the direct effects that selenium has on carcinogenesis from its impact on the growth of established tumors as well as its influence on cancer immunity. One of the direct anti-cancer effects of selenium is related to the ability of seleno-compounds to induce oxidative stress and DNA damage accumulation and, consequently, apoptosis [15]. Other direct effects of selenium on established tumors in humans are less clear and this is particularly true for those effects that are exerted through the immune system. For example, there is some evidence from one human study suggesting an inhibitory effect of selenium on the epithelial-to-mesenchymal transition (EMT) that drives metastasis [157]. This was accompanied by the capacity of higher levels of selenium to down-regulate expression of genes involved in wound healing and inflammation, which are both related to EMT. The idea that selenium supplementation may be used to support the immune system during cancer treatment has been supported by some studies including those related to childhood leukemia and neutropenia [158,159]. Intervention studies showed positive effects of selenium on mitigating neutropenia in children suffering from leukemia/lymphomas as well as solid tumors [160].

There is evidence that GPX4 modulates hepatocellular carcinoma (HCC) in both humans and rodent models. In humans, GPX4 expression in tumors positively correlated with patient survival and was linked to pathways that regulate cell proliferation, motility, tissue remodeling, and immune responses with a particular effect on M1 macrophage polarization [161]. Corroborative results demonstrate that overexpression of GPX4 decreased the growth of human HCC cell lines using xenotransplantation into immune-deficient non-diabetic (NOD) mice. These findings are consistent with previous studies showing that inhibition of GPX4 expression by siRNA in HCC cells increased the formation of Vascular endothelial growth factor (VEGF) and IL-8 cytokines [162], which are both clinically relevant adverse prognostic factors in HCC patients [162,163]. However, since NOD mice do not include a competent immune system, it is difficult to interpret how the immune relevant data from the human gene arrays can be related to the rodent studies.

The polarization of tumor-associated macrophages away from tolerogenic phenotypes and toward anti-tumor M1 macrophages suggested in the above experiments with GPX4 overexpression was also supported in selenium nanoparticle studies [164]. However, how selenium levels affect macrophage polarization in the tumor microenvironment in human cancers remains to be determined. As discussed in a previous review [165], higher levels of selenium can increase NK cell activity by preventing the non-enzymatic formation of parafibrin that surrounds tumor cells and hinders immune surveillance and by activating the NK cell population in the tumor microenvironment. The anti-tumoral activity of NK cells requires the expression of the activating receptor natural killer group 2 member D (NKG2D) on NK cell surfaces [166]. The selenium metabolite known as methylselenol was found to upregulate two NKG2D ligands on the surface of tumor cells [167]. However, it was not determined if this led to increased NK cell killing of tumor cells. This feature is important for the detection of tumor cells by CD8^+^ T cells since these cells also express NKG2D. In fact, major histocompatibility-I (MHC-I) present tumor antigens to CD8^+^ T cells to activate their cytotoxic activities, which is also affected by methylselenol in cancer cells. In particular, this selenium metabolite was shown to alter redox metabolism in melanoma cells and lead to increased levels of MHC-I cell surface antigens [168]. This study showed that the actions of methylselenol mimic IFNγ signaling by also upregulating members of IFNγ responsive genes. However, one must consider the detrimental effects of inducing oxidative stress in some tissues such as the gut where this can promote tumorigenesis and tumor progression [85].

Due to the ability to control experimental conditions, rodent models of selenium and cancer have provided data that may be easier to interpret. However, unless specifically built into the study design, it is difficult if not impossible to distinguish between the effects of the bioavailable selenium on the cancer cells themselves versus the immune cells that are either trying to facilitate tumor progression or trying to eradicate the cancer cells. The mixed results for rodent cancer studies when tumor growth is the primary endpoint for mouse studies highlight this confounding issue. For example, in our mouse model study of syngeneic mesothelioma tumors that utilized immune competent animals, we expected that increasing dietary selenium would hinder tumor progression due to enhanced anti-cancer immunity. However, the tumors progressed at an accelerated rate in mice that were fed higher selenium diets due to the pro-reducing capacity in the tumor cells themselves [169]. Other rodent studies focused on melanoma or breast cancer found different results with higher selenium intake leading to lower tumor growth concurrent with immune enhancing effects [170]. When immune responses have been analyzed, the predominant effect is an enhancement of Th1 immunity and a reduction in regulatory T cells (Tregs) and myeloid-derived suppressor cells that suppress anti-tumor immunity [171,172]. There are many factors to consider when analyzing the results of the rodent cancer studies including the type of cancer, strain, and immune status of the rodents, dose and form of selenium used, and endpoints used for analyses. The generation of new xenograft models as well as humanized rodents will facilitate these studies when the research field moves forward.

## 6. Specific Mechanisms by Which Selenoproteins Regulate Immunity

There have been some investigations into molecular mechanisms underlying the effects of selenium on the immune system. Because selenium can impact so many cellular functions, it is very difficult to dissect out the many different pathways and individual molecules regulated by this micronutrient. Despite these caveats, the generation of transgenic and knockout mouse models has revealed some intriguing mechanisms involving individual selenoproteins. Two examples shown below involve roles elucidated for two selenoproteins in regulating immune cell functions.

MSRB1 is the lone Sec-containing member of the methionine sulfoxide reductase (MSR) family of proteins, which also includes MSRA, MSRB2, and MSRB3. Reactive oxygen species (ROS) can oxidize methionine residues in proteins to produce a mixture of *S*-stereoisomers and *R*-stereoisomers of methionine sulfoxide, which are reduced by MSRA and MSRB enzymes, respectively [173]. The first report of MSRB1 in macrophage biology described its role in regulating actin polymerization during cellular activation that promoted functions like phagocytosis and cytokine secretion [174]. Two methionine residues in actin are specifically converted to methionine-R-sulfoxide by monooxygenase enzymes called Mical1 and Mical2 and are reduced back to methionine by selenoprotein MSRB1, which supports actin disassembly and assembly, respectively. In this manner, macrophages utilize a system of redox reactions during cellular activation by stimulating MSRB1 expression and activity as a part of innate immunity. There are two intriguing aspects of this regulatory process. First, lipopolysaccharide (LPS) induces expression of MSRB1, but not other MSRs, which suggests stereo-specificity in the reactions. Second, a follow-up study found that MSRB1 controls immune responses by promoting anti-inflammatory cytokine expression in macrophages [35]. It must be noted that bacteria have their own versions of MSRs and the importance of this was shown in the case of *Francisella tularensis*, which replicates within macrophages during infection. The *F. tularensis* MSRB (the only B isoform in bacteria) was crucial in promoting replication while MSRA was not as important [175]. In fact, the roles of MSRB in the pathogenesis of several bacteria have been investigated, but it must be noted that the bacterial enzyme contains cysteine (Cys) in place of Sec and, therefore, these MSRBs are not selenoproteins. Overall, it remains to be determined how the regulated expression of MSRB1 in humans affects infection with this bacterium or other pathogens that replicate within macrophages, but this selenoprotein appears to be quite important in shaping innate immune responses.

Our laboratory has studied SELENOK in the immune system for a number of years starting with the finding that levels of this selenoprotein were relatively high in immune tissues of mice and its expression increased with higher selenium intake [42]. SELENOK knockout mice showed no overt phenotype and were fertile, but we found ~50% reduction in most immune cell functions when the immune system was challenged [42]. We subsequently identified its role in promoting calcium flux during immune cell activation by SELENOK complexing with the enzyme DHHC6 (letters represent the amino acids aspartic acid, histidine, histidine, and cysteine in the catalytic domain and 6 represents the 6th member of the family) to palmitoylate to the endoplasmic reticulum (ER) calcium channel protein inositol-1,4,5-trisphosphate receptor (IP3R) [176]. SELENOK itself does not function as an enzyme but instead binds to DHHC6 to stabilize the acylated intermediate of this enzyme so that it is not hydrolyzed by water, which does not hydrolyze the thioester bond between the acyl group and the cysteine residue in DHHC6 before it can transfer the acyl group to target proteins like IP3R [177]. Other proteins involved in immune functions also depend on SELENOK/DHHC6 for palmitoylation to carry out their activities including CD36 and Arf-GAP with SH3 domain, ANK repeat and PH domain-containing protein 2 (ASAP2) [178,179]. Overall, SELENOK plays an important, non-enzymatic role in regulating immunity by functioning as a cofactor for an enzyme involved in critical post-translational modifications of proteins.

## 7. Conclusions

The immune system is one aspect of human health that is impacted by dietary selenium levels and selenoprotein expression. Under conditions of selenium deficiency, innate and adaptive immune responses are impaired. The benefits of selenium supplementation to boost immunity against pathogens, vaccinations, or cancers have been explored and have not provided entirely clear results. Some of the issues lie in the fact that some pathogens or tumor cells may themselves benefit from higher levels of selenium. Manipulation of individual selenoproteins may offer a more precise approach for enhancing the immune system or mitigating chronic inflammation. This approach will require a comprehensive characterization of the roles for selenoproteins and an unmasking of molecular mechanisms by which they regulate immune cell functions.

## Figures and Tables

**Table 1:** Summary of Selenoprotein Functions.

Selenoprotein	Abbreviations	Functions (References)
Glutathione peroxidase 1	GPX1, cytosolic glutathione peroxidase	Reduces cellular H_2_O_2_ [22,23].
Glutathione peroxidase 2	GPX2, intestinal glutathione peroxidase	Reduces peroxide in gut [24,25].
Glutathione peroxidase 3	GPX3, Plasma glutathione peroxidase	Reduces peroxide in blood [26,27].
Glutathione peroxidase 4	GPX4, Phospholipid hydroperoxide glutathione peroxidase	Anti-oxidative lipid repair enzyme localized to cytosol, mitochondria, and nucleus, which reduces hydrogen peroxide radicals and lipid peroxides to water and lipid alcohols and prevents iron-induced cellular ferroptosis [28,29].
Glutathione peroxidase 6	GPX6	Importance unknown [30].
Thioredoxin reductase 1	TXNRD1, TR1	Localized to cytoplasm and nucleus and regenerates reduced thioredoxin [31].
Thioredoxin reductase 2	TXNRD2, TR3	Localized to mitochondria and regenerates reduced thioredoxin [32].
Thioredoxin-glutathione reductase	TXNRD3, TR2, TGR	Testes-specific expression, which regenerates reduced thioredoxin [33].
Iodothyronine deiodinase 1	DIO1, D1	Important for systemic active thyroid hormone levels [34].
Iodothyronine deiodinase 2	DIO2, D2	ER enzyme important for local active thyroid hormone levels [34].
Iodothyronine deiodinase 3	DIO3, D3	Inactivates thyroid hormone [34].
Methionine-R-sulfoxide reductase B1	MSRB1, SELR, SELX	Regulator of F-actin repolymerization in macrophages during innate immune response, which works in concert with MICALs to reduce oxidated methionine (R)-sulfoxide (Met-RO) back to methionine [35,36].
Selenoprotein F	SELENOF, Selenoprotein 15, SEP15	ER-resident thioredoxin-like oxidoreductase that complexes with uridine-guanosine-guanosine-thymodine (UGGT) and improves protein quality control by correcting misglycosylated/misfolded glycoproteins via the calnexin-calreticulin- endoplasmic reticulum proten 57 (ERp57) axis and pH-dependent endoplasmic reticulum proten 44 (ERp44 )system [37,38].
Selenoprotein H	SELENOH, SELH, C11orf31	Nuclear localization, which is involved in redox sensing and transcription [39,40].
Selenoprotein I	SELENOI, SELI, EPT1	Involved in phospholipid biosynthesis [41].
Selenoprotein K	SELENOK, SELK	Transmembrane protein localized to the endoplasmic reticulum (ER) and involved in calcium flux in immune cells and ER associated degradation in cell lines [42,43].
Selenoprotein M	SELENOM, SELM, SEPM	Thioredoxin-like ER-resident protein that may be involved in the regulation of body weight and energy metabolism [44].
Selenoprotein N	SELENON, SELN, SEPN1	Transmembrane protein localized to ER. Mutations lead to multiminicore disease and other myopathies [45,46].
Selenoprotein O	SELENOO, SELO	Mitochondrial protein that contains a C-X-X-U (where C is cytosine, X is any nucleotide, and U is uridine) motif suggestive of the redox function [47].
Selenoprotein P	SELENOP, SEPP1, SeP, SELP, SEPP	Secreted into plasma for selenium transport to tissues [20,48].
Selenoprotein S	SELENOS, SELS, SEPS1, VIMP	Transmembrane protein found in ER involved in ER associated degradation [49,50].
Selenoprotein T	SELENOT, SELT	Oxidoreductase localized to the Golgi complex and ER and manifests a thioredoxin-like fold and is involved in redox regulation and cell anchorage. Complexes with UGGTs to improve PQC. Deficiency leads to early embryonic lethality [51].
Selenoprotein V	SELENOV, SELV	Testes-specific expression [21].
Selenoprotein W	SELENOW, SELW, SEPW1	Putative antioxidant role, which may be important in muscle growth [52].
Selenophosphate synthetase 2	SEPHS2, SPS2	Involved in synthesis of all selenoproteins including itself [53].
